# Does Internet Use Connect Us to a Healthy Diet? Evidence from Rural China

**DOI:** 10.3390/nu14132630

**Published:** 2022-06-24

**Authors:** Baojie Ma, Xin Jin

**Affiliations:** China Academy for Rural Development (CARD), Zhejiang University, Hangzhou 310012, China; mabaojie@zju.edu.cn

**Keywords:** internet use, dietary quality, rural China, fixed effect, instrumental variable

## Abstract

Dietary patterns in China have changed dramatically over the past few decades as the Internet has become rapidly available. Based on data from the China Health and Nutrition Survey (2006–2011), we use a two-way fixed effects model and an instrumental variable approach to determine the impact of Internet use on the dietary quality of rural residents. The results indicate that Internet use could significantly improve the dietary quality of Chinese rural residents, with an increase of about 10.4% in the China Food Pagoda Score (CFPS), mainly due to the increase in the dietary quality score for five food groups: fruits, meats, eggs, oil, and salt. We also found that Internet use significantly increased the consumption amounts of milk and its products (4 g), fruits (31 g), eggs (8 g), and vegetables (34 g), while also decreasing the intake of salts (2 g) and oil (6 g). A possible mechanism is that Internet use improves the dietary knowledge of rural residents, thus optimizing their dietary structure. Moreover, the effect of the Internet was greater among females and those who prepare food for a family. Rural residents without a college degree enjoyed more benefits. In summary, governments should further promote Internet penetration in rural areas for health purposes.

## 1. Introduction

The improvement in nutritional status as an important element of the Sustainable Development Goals 2.0 (SDG 2.0) plays an indispensable role in reducing poverty and inequality [1,2].

Nutrition improvement involves two aspects, namely reducing malnutrition and avoiding overnutrition. The occurrence of the former can lead to stunting, illness, and even death in children [3], while the latter can lead to an epidemic of overweight obesity [4,5]. Especially in some low- and middle-income countries, the economic cost of overweight obesity will be as high as USD 7 trillion in the next 15 years [6]. Therefore, improving nutrition is of great importance from both health and economic perspectives.

China’s economy and agriculture have grown rapidly over the past decades, which has brought about a significant shift in the dietary patterns of domestic consumers. A large body of literature suggests that both urban and rural residents in China are experiencing a shift from the traditional low-fat Chinese diet, characterized by cereals and vegetables, to a Western-style diet, which is characterized by high amounts of calories, fat, and sugar [7,8,9,10,11].

These changes in dietary patterns have had a significant impact on the nutritional and health status of the Chinese population. On the one hand, the prevalence of undernutrition in China has declined significantly with the increase in food consumption. According to estimates by the International Food and Agriculture Organization (FAO), the prevalence of undernutrition in China has decreased from 24% in the early 1990s to less than 10% in recent years [12]. On the other hand, due to the structural changes in diet, some highly processed foods that are high in fat and sugar have become overconsumed, and this has led to the occurrence of non-communicable diseases such as diabetes and overweight obesity [13,14]. To address the coexistence of undernutrition and overnutrition and to guide residents toward a healthy diet, the National Health and Family Planning Commission of the People’s Republic of China (NHFPC) released the 2016 China Dietary Guidelines (CDG2016), which provide specific dietary recommendations for all healthy people over the age of two years in China.

Many scholars are aware of the importance of improving the diet of the population, and a significant body of literature has been published to reveal the factors influencing diet quality, including urbanization [15], income [7], family food environment [16], food accessibility [17], female empowerment [18], educational attainment [19], specialization of agricultural production [20], etc. However, studies on how Internet use affects diet quality are rare.

China has experienced a period of rapid Internet development in the past two decades, and it now has the largest number of Internet users in the world. By June 2021, China’s Internet penetration rate had reached 71.6% according to the China Internet Network Information Center (CNNIC), and the Internet has profoundly affected the lives of rural residents in China [21]. Theoretically, the Internet reduces the cost of information transmission [22] and broadens users’ information channels, which may help residents improve their dietary knowledge and thus make healthier dietary consumption choices. In Uganda, cellphone use is positively associated with household dietary diversity. Moreover, compared to men, women using cellphones can provide a stronger improvement [23]. The author attributes this to the fact that cellphone use increases household income and promotes gender equality, both of which positively affect food diversity. Conditions in Kenya are very similar, and there is an indication that cellphone use can improve dietary diversity, since the cellphones facilitate the purchasing of different kinds of foods [24,25]. Internet use can also positively act on dietary quality by other pathways. For example, the Internet reduces transaction costs in agricultural markets and helps farmers to participate in the market, thus providing more types of agricultural products [26], which helps to increase the food accessibility of consumers and ultimately improve their dietary quality [27,28]. Based on this judgment, the government, from the perspective of improving the dietary quality and health of the residents, should increase investment in Internet infrastructure, thus further promoting the popularity of the Internet.

However, some scholars have argued that the relationship between the Internet and dietary quality should be judged with more caution since health information on the Internet may mislead residents [29,30], thus causing them to make unhealthy food choices (e.g., dieting), with a resulting deterioration in their diet quality. In addition, the use of the Internet may change people’s dietary preferences, increase people’s preference for high-calorie foods and caloric intake [31,32], which may also adversely affect their dietary quality. In this case, the government’s investment in Internet infrastructure needs to be more thoroughly considered.

Based on three waves (in 2006, 2009, and 2011) of nationally representative tracking surveys in China, this paper empirically investigates the effect of Internet use on diet quality by taking the Internet use of rural residents as an example to answer the following three questions: How does the dietary quality of rural residents who use the Internet change compared to those who do not use the Internet? What are the mechanisms by which Internet use causes changes in dietary quality? Are the effects of the Internet heterogeneous between different groups?

Following recent research [33] which found that Internet access can significantly contribute to rural residents’ caloric and protein daily intake, we make four main contributions in this study. First, instead of simply analyzing nutritional intake (i.e., fat, calories, and protein), we constructed a Chinese dietary health score (CFPS) to comprehensively evaluate the relationship between the Internet use and dietary quality. Second, panel data was used in this research in order that a two-way fixed effects model could be applied in our empirical design that would prevent individual-level non-time-varying confounding factors from interfering with the results. Additionally, the instrumental variable approach was also used to handle potential endogeneity, which ensured the robustness of our results. Third, food consumed away from home was fully considered in our data, which effectively filling in the gaps in other studies. Lastly, the possible mechanism—that Internet use enriches users’ dietary knowledge—was empirically tested, which could reveal more implications.

The remainder of the article is organized as follows. Section 2 provides the materials and methods. Section 3 introduces the empirical results. Some discussions are reported in Section 4. Section 5 concludes our paper.

## 2. Materials and Methods

### 2.1. Empirical Methods

This paper constructs a two-way fixed effects model to investigate the impact of Internet use on dietary patterns. The specific mathematical model is as follows:

(1) $$Y_{ict}=\rho_{0}+\rho_{1}internet_{ict}+P_{ict}{}^{T}\alpha +C_{ct}{}^{T}\omega +\delta_{i}+\gamma_{t}+\varepsilon_{ict}$$

where *i* denotes the individual, *c* denotes the village, and *t* denotes the year. $Y_{ict}$ denotes the dietary quality of individual *i* located in village *c* in year *t*. $internet_{ict}$ is the core independent variable of interest in this paper, which is a dummy variable assigned to 1 if respondent *i* uses the Internet in year *t*, and 0 otherwise. $P_{ict}$ is a matrix of control variables reflecting the characteristics of individuals and their households, including respondent age, education level, total household income, household size, the proportion of household members engaging in agricultural production, the proportion of household members who leave the home for work, work intensity, and whether there is a refrigerator, a pressure cooker, a car, or a motorcycle in the household. $C_{ct}$ is a matrix of control variables reflecting the characteristics of the village, including whether it is connected to a paved road and the number of free markets. To prevent each individual’s non-time-varying factors (e.g., gender) from interfering with the results, we controlled for individual-fixed effects, denoted by $\delta_{i}$. Moreover, to prevent macro factors within the national level from interfering with the results (e.g., national GDP), we also controlled for year-fixed effects, denoted by $\gamma_{t}$. $\varepsilon_{ict}$ denotes the random error term.

### 2.2. Data Collection

The data used in this paper are from the three-wave panel data of the China Health and Nutrition Survey (CHNS) of the University of North Carolina Population Center in 2006, 2009, and 2011 (To date, CHNS is the only nationally representative database that can provide detailed information on food consumption of Chinese residents. Although the CHNS database has been updated to 2015, the detailed food intake (i.e., each food group) information of respondents is not available in CHNS 2015 yet. This means that this study and some other recent studies can only utilize the CHNS data up to 2011). Samples in CHNS were selected using multi-stage random sampling in nine provinces of China (e.g., Guangxi, Guizhou, Henan, Heilongjiang, Hubei, Hunan, Jiangsu, Liaoning, and Shandong). Detailed information can be found in the literature [34,35]. This database is representative of the total population of China and has been widely used in current academic research, including in research published in several authoritative journals (e.g., [11,17]).

**Figure A1:**
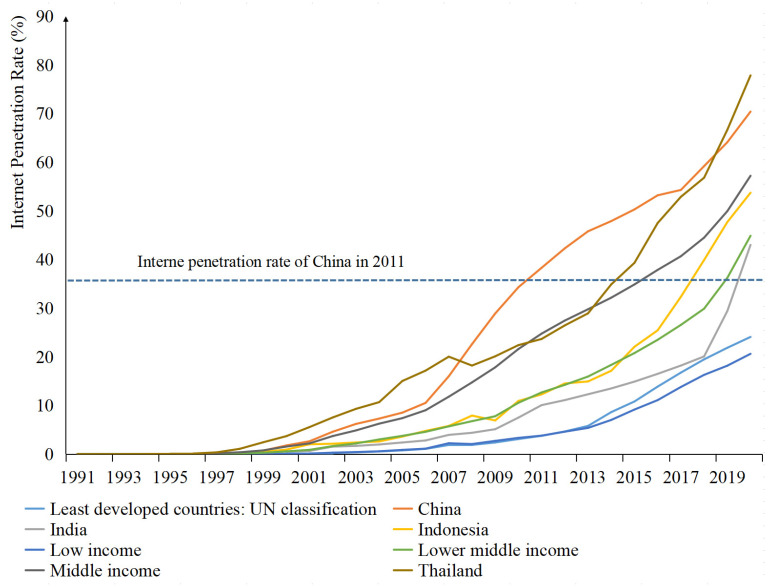
Internet penetration rate in some countries (regions) from 1991–2020. *Source*: World Bank database, https://data.worldbank.org/indicator/IT.NET.USER.ZS (accessed on 1 March 2022).

The data in CHNS can adequately support our study. On the one hand, according to Figure A1 (Appendix A), China’s Internet penetration rate in 2011 was even higher than that of many countries or regions in 2017 (Countries: India, Indonesia. Regions: LDCs, low-income countries, and lower middle countries). Therefore, although our data is 10 years old, it can still provide many policy and research implications for other developing countries. On the other hand, at the individual and household levels, detailed information about the basic socioeconomic situation of individuals, dietary structure, and basic household conditions are provided. Moreover, at the village level, information about village infrastructure conditions, services, and population are also provided. In this paper, we focus on the effect of Internet use on the dietary quality of rural adults, and we excluded individuals in the sample who are under 18 years old and over 60 years old. To avoid the effect of outliers on the results, responders with a total calorie intake lower than 5% or higher than 95% of the sample were excluded. Finally, to ensure the validity of the two-way fixed effects model, individuals who appeared only once during the survey were also removed.

### 2.3. Measurement of Key Variables

#### 2.3.1. Dependent Variable: Dietary Quality

The dependent variable in this paper is the respondent’s diet quality. CHNS obtains data on individuals’ three meals per day, including meals eaten in the house and meals eaten away from home, for three random days in the past week using the 24 h Personal Diet Questionnaire. In addition, CHNS also records changes in household food inventories, which are used to measure individual oil and salt consumption. After counting the number of meals eaten by all household members, the daily consumption of each food group as well as the consumption of salt and oil per person is calculated. In addition, CHNS also considers the dining-out situation of respondents in the process of data collection, and the consumption of various foods when dining out is recorded.

In accordance with the Chinese Food Pagoda 2016 (CFP2016), we divided the food consumed by the respondents into a total of ten groups. China Dietary Guidelines 2016 (CDG2016) is jointly presented by the Chinese Center for Disease Control and Prevention (CDC), the National Health and Family Planning Commission of the People’s Republic of China, and the Chinese Nutrition Society [13]. The CFP2016 provides the recommended daily consumption for healthy Chinese adults for five major food groups in accordance with the main principles proposed by the CDG2016. In addition, the CFP2016 provides specific values for the upper and lower intakes of ten food groups, as detailed in column (1) of Table A1 (Appendix A).

Our key dependent variable, the Chinese Food Pagoda Score (CFPS), was calculated according to the method in a recent study [17]. Each food group is assigned a score of 1, 0.5, or 0. Specifically, the score denotes “1” if the real consumption of a food group is within the recommended consumption interval, and a score of 0.5 is assigned when the actual intake of a food group is 50% higher than the upper bound or 50% lower than the lower bound. If the actual intake of a food group differs significantly from the recommended value, the score is “0”. It is important to note that no upper bound is set for fruits or vegetables in order to encourage healthy eating behavior. The CFPS of each respondent is obtained by summing up the scores of these 10 food groups, and the higher the CFPS, the closer the respondent’s dietary structure is to the CFP2016 recommendations, and the higher the dietary quality is. The specific assignment methods are shown in columns (2)–(8) of Table A1 (Appendix A).

#### 2.3.2. Core Independent and Control Variables

The main independent variable that is the focus of this paper is whether the respondents use the Internet. If the respondents use the Internet, we assigned a value of 1 to the Internet dummy; otherwise, the value is 0.

In terms of control variables, we controlled for responders’ age and its square, because the probability of Internet use may also change with age increasing. Furthermore, age is often seen as a variable that affects diet quality. Years of education were also used as a control variable. Internet use has a certain learning cost, and as respondent education increases, a corresponding decrease in learning costs occurs; in addition, their likelihood of using the Internet may increase, and people with higher education levels appear to have a healthier diet.

In order to exclude possible interference of labor intensity on diet quality, this paper also includes variables reflecting respondents’ labor intensity, which are reflected by ordered numbers 1–5 in the CHNS questionnaire. Household appliances, cooking utensils, and transportation play important roles in storing food, preparing food, and transporting food in the household, respectively, so we also controlled for refrigerator ownership, pressure cooker ownership, car and motorcycle ownership in each respondent’s household. Some household characteristics were also included, that is, household size, total household income (log-form), the proportion of household members engaging in agricultural production, and the proportion of household members who leave the home for work. At the village level, considering that some transportation infrastructure may have an impact on food market supply and thus on residents’ food consumption, whether the village is connected to a paved road was included as a control variable. In addition, food accessibility is also an important factor that may affect food consumption; we thus controlled for the number of free markets in the village as well.

#### 2.3.3. Descriptive Statistics

**Figure 1:**
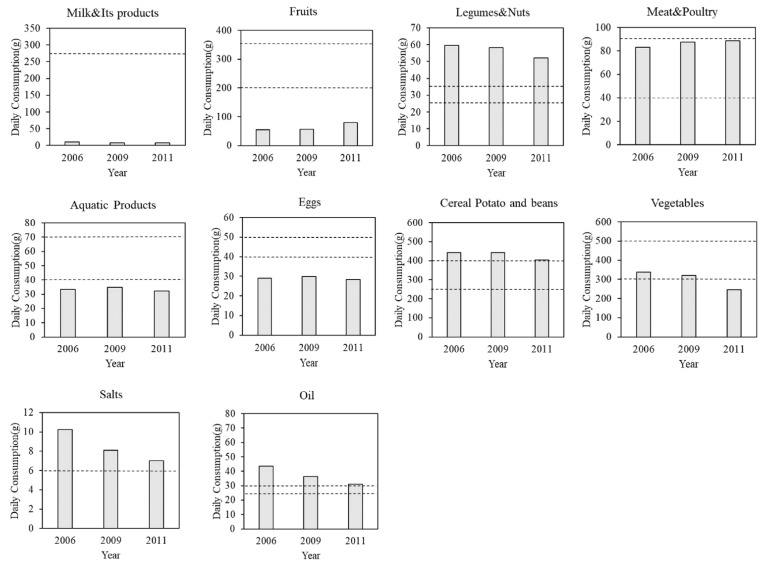
Actual consumption of various food groups by rural residents in China (2006–2011). *Source*: Author calculated by CHNS.

This paper first describes the consumption of various food groups by rural residents in the sample from 2006 to 2011 and compares it with the recommended amounts from CFP16 (Figure 1). The trend shows that the consumption of fruits as well as meat and poultry increased significantly from 2006 to 2011, but fruit consumption remained well below the recommended amount. The consumption of milk, aquatic products, and eggs showed a moderate rise, but all of them were consumed less often than the lower bound of the recommended amount as well. As for nuts, cereals, potatoes, beans, vegetables, oil, and salt, their consumption in rural areas showed a downward trend. In addition, the consumption of nuts, salts, and oil was far higher than the recommended upper bound. The consumption of cereals, potatoes, and beans by rural residents, on the other hand, began to fall almost into the recommended range. At the same time, however, vegetable intake began to fall below the CFP16 recommendations.

Table 1 provides descriptive statistics for the main variables of this paper. The average CFPS of rural adults was 3.19, which was less than half of the full score. Their Internet usage rate was relatively low, at only 7% (China’s rural Internet penetration rate in 2009 was 7.1% (CNNIC, 2010)). The average age of the respondents was 45 years old, mainly middle-aged rural residents. The average education level was between primary and junior high school. The average household size was 4.01 persons. The average labor intensity by the respondents was relatively moderate. A total of 60% and 51% of the respondents owned a refrigerator and a pressure cooker, respectively. There were 8% and 48% rural residents who had cars and motorcycles, respectively. On average, about 43.15% of the respondents’ household members were engaged in agriculture, and about 1/3 of the respondents’ household members left the home for work. At the village level, 72% of the villages were connected to paved roads. Moreover, there were 2.88 free markets in each village, on average.

## 3. Results

### 3.1. Baseline

The impact of Internet use on the CFPS of Chinese residents is presented in Table 2. In this paper, we introduced control variables stepwise to test the robustness of the coefficients. In column (1), no control variables were included. Moreover, in column (2), we introduced individual-level control variables. In column (3), village characteristics were also added. The coefficients of the independent variables did not change significantly during the above process, proving the robustness of our conclusions. In column (3), the results demonstrate that Internet use positively impacted the dietary quality of rural residents, and the coefficient was statistically significant at the 1% level. Specifically, Internet use increased the CFPS by 0.33, which was about 10.4% compared with the mean value.

In addition, the individual and community characteristics also had impacts on dietary quality. For example, rural responders’ CFPS increased significantly with age square. A larger household size, on the other hand, was detrimental to CFPS. As for household assets, respondents with a refrigerator in the household had a higher quality of diet. The higher the proportion of household members engaged in agricultural production, the higher the quality of rural adults’ diets. The number of free markets in a village was significantly positively correlated with the quality of the diets of the respondents in that village.

### 3.2. Further Exploration: Effects on Score of Each Food Group

In this section, we further explore the effect of Internet use on the CFPS scores for 10 categories of food separately. The results are shown in Table 3.

We find that Internet use had a positive impact on the CFPS scores for five food groups: fruits, meats, eggs, oil, and salt. Their CFPS scores improved by 0.10, 0.08, 0.08, 0.15, and 0.10, respectively. It is important to bear in mind that all dependent variables in Table 3 were made up of the numbers 0, 0.5, and 1. Therefore, the real impact was relatively large for most food items.

We also used the real consumption of each food group as the dependent variable and estimated the impact of Internet use on the rural adults’ various food intake. The results are represented in Table 4.

The results demonstrate that Internet use led to a significant increase in the consumption of milk and its products by 4 g (207% compared to the mean value), fruits by 31.4 g (76% compared to the mean value), eggs by 7.7 g (30% compared to the mean), and vegetables by 33.7 g (12% compared to the mean). By contrast, the use of the Internet also significantly reduced the daily oil and salt consumption of rural adults by 6 g (18% compared to the mean) and 1.9 g (21.86% compared to the mean), respectively.

### 3.3. Mechanism Analysis: The Level of Dietary Knowledge Is Improved after Using the Internet

Our study examined potential pathways linking Internet use to improved dietary outcomes in this section. Due to the use of the Internet, the rich information resources provided by Internet platforms can make it easy for people to acquire health-related information in a short time, which may motivate people to make healthier food consumption decisions.

To test the above speculation, this paper verified the relationship between Internet use and the level of dietary knowledge of rural adults. CHNS has been paying attention to the dietary knowledge of respondents aged over 12 years since 2004. A nine-item quiz on basic dietary knowledge was given to respondents, as reported in Table A2 (Appendix A). For each question, respondents chose from “strongly agree”, “fairly agree”, “neutral”, “fairly disagree”, “strongly disagree”, and “unknown”. Based on the WHO (1998) criteria, we created an indicator in which a value of 1 denotes a correct answer, 0 stands for “unknown”, and −1 for an incorrect answer. We then constructed a summary index “Diet Knowledge Score (DKS)” (Range from −9 to 9) from these responses [36].

The impacts of Internet use on the dietary knowledge of rural adults are shown in column (1) of Table 5. The results reveal that the use of the Internet led to a 0.50 increase in rural adults’ DKS, and the coefficient was significant at the 10% level. Considering that the mean value of the DKS in our samples was 4.29, the use of the Internet could lead to an increase of 11% in dietary knowledge compared to the mean value. In column (2), a dummy variable was also selected to reflect rural adults’ dietary knowledge level, that is, whether the responders knew the Chinese dietary pagoda. The results indicate that Internet use also led to a significant increase of 40% in the probability that rural adults would know about the Chinese dietary pagoda. Furthermore, in columns (3) and (4), we verified whether an increase in dietary knowledge could improve the dietary quality of rural adults, and both coefficients of dietary knowledge were positive and significant.

In summary, the use of the Internet has provided rural residents with a wealth of health information and dietary knowledge, thus helping them to understand what a healthy dietary structure is. Therefore, as the level of dietary knowledge of rural residents improves, they will make more optimal and healthier food consumption choices to improve the quality of their diets.

### 3.4. Heterogeneity Effect

Considering that there are relatively large differences among various rural residents, the improvement effect of the Internet on diet quality may likewise show heterogeneity among them. To verify this heterogeneity, based on the baseline regression (Equation (1)), we introduced interaction terms with the Internet and individual characteristics, the results are shown in Table 6.

First, in column (1) of Table 6, we compared the heterogeneity of the impact of Internet use between male and female rural residents. We set a dummy variable “Female” which takes the value 1 if the responder is female, and 0 otherwise. The coefficient of the interaction term between Female and Internet was 0.27, and it was significant at the 10% level. This indicates that the dietary improvement effect of the Internet on females was significantly greater than on males by 0.27. This result is consistent with previous research [23], and the potential logical explanation behind this can be attributed to women are more likely to seek online health information than men [37].

In column (2), we investigated whether the impact of Internet use is different between residents who mainly prepare food for the family. A dummy variable “Cook” was assigned to 1 if the responder is the person mainly responsible for cooking in the family, and 0 otherwise. The coefficient of interaction term between Cook and Internet was significantly positive, indicating that the improvement effect of the Internet on dietary quality was stronger if the Internet user was the person who mainly prepared food for their family.

In column (3), we compared the heterogeneity effects of Internet use among rural residents with relatively high and relatively low education. A dummy “High edu” was assigned to 1 if the responder has received a college or higher degree, and 0 otherwise. The coefficient of interaction term was significantly negative, demonstrating that the Internet had a greater effect on rural residents with low educational attainment than rural residents with high educational attainment. It is important to note that the mean CFPS was slightly higher for the highly educated respondents; the above results therefore imply that Internet use can reduce the gaps in dietary quality between different educated groups.

Finally, we also explored the effect on high-income individuals and low-income individuals, as reported in column (4). The dummy variable “High income” takes the value 1 if the responder’s income is higher than the median of the sample in the current year; otherwise, it takes the value 0. We found that the coefficient of the interaction term was insignificant, which means that there is no statistical difference in the Internet use effect between high-income individuals and low-income individuals.

### 3.5. Robustness Check

#### 3.5.1. Handling Endogeneity Issues

In this study, we used longitudinal data and a two-way fixed effects model, which allow for stronger causal claims about the relationship between Internet use and dietary quality outcomes than cross-sectional data [38]. However, some unobservable time-varying variables that affect both the quality of the respondent’s diet and the Internet use behavior may cause endogenous problems and lead to biased results. For example, if a respondent pays more attention to a healthy lifestyle, the quality of his or her diet will be correspondingly higher. At the same time, a health-conscious attitude will also motivate the respondent to seek more information sources to obtain health-related information, and he or she will have a stronger incentive to use the Internet. Under this assumption, the baseline fixed effects model would overestimate the positive effect of the Internet on dietary quality.

In order to exclude the endogeneity, the two-stage least squares (2SLS) estimation was used in this paper. The specific mathematical form was as follows:

(2) $$\mathrm{First}\;\mathrm{Stage}:Internet_{ict}=\beta_{0}+\beta_{1}IV_{ct}+P_{ict}{}^{\prime }\alpha +C_{ct}{}^{\prime }\omega +\delta_{i}+\gamma_{t}+\varepsilon_{ict}$$

(3) $$\mathrm{Second}\;\mathrm{Stage}:Y_{ict}=\omega_{0}+m_{1}\hat{Internet_{ict}}+P_{ict}{}^{\prime }\tilde{\alpha }+C_{ct}{}^{\prime }\tilde{\omega }+\tilde{\delta_{i}}+\tilde{\gamma_{t}}+\varepsilon_{ict}$$

where $IV_{ct}$ was our instrumental variable (IV), and the other variables were defined as above. We examined whether the respondent had a computer at home and considered this as the IV, which is widely used as an instrumental variable for the Internet [39].

A good IV needs to be highly correlated with the endogenous variables while being uncorrelated with the residual term [40]. On the one hand, Broadband (computer) is the main way for Chinese to access the Internet until 4G (smartphone) became popular in 2013. Therefore, whether people had a computer from 2006 to 2011 is strongly and positively associated with their Internet use. On the other hand, whether there was a computer at home did not directly affect people’s diet or indirectly affect their diet through channels other than the Internet.

The regression results of the 2SLS approach are reported in Table 7. The bottom half of Table 7 shows the first-stage regression results. It indicates that the instrumental variable had a positive and significant effect on the rural responders’ Internet use. Additionally, since the F-value of the first stage regression was greater than 10, the hypothesis of weak instrumental variable could be rejected. Moreover, the results of the second-stage regression (upper half of Table 7) were similar to those in our baseline regression, which shows that Internet use led to a significant increase in rural residents’ CFPS by 0.79–0.90 (25–28% compared to the mean value).

#### 3.5.2. Changing Independent Variable to Some Other Healthy Dietary Variables

Two types of foods that may pose a threat to a healthy diet that were not considered above are sugared drinks and alcohol. Some studies have pointed out that sugared drinks are a significant contributor to current childhood and adult obesity [41,42]. Moreover, alcohol is also widely considered as a predisposing factor for many types of cancer [43]. Therefore, we further examined whether Internet use affects the intake of these two food groups among rural residents as the robustness check. The results are shown in Table 8.

Column (1) of Table 8 shows that Internet use significantly increased the consumption of sugary beverages among rural residents, which may be related to the fact that users see more advertisements about soft drinks online [44]. The results in columns (2) and (3), on the other hand, indicate that Internet use to some extent lowered the alcohol intake of rural residents, but both coefficients had relatively large standard errors, and were thus at insignificant levels.

## 4. Discussion

The dietary patterns of Chinese residents have changed significantly, especially in the last two decades, which tended toward a westernization charactered by diets with high fat and sugar [7,8,9,10,11]. During the same period, Internet technology also gained rapid popularity and application in China. It is urgent to explore the relationship between the Internet use and dietary quality. Therefore, we investigated the impact of Internet use on the dietary quality of rural adults in this study.

By comparing with the recommended food intake quantity in CFP16, we constructed a Chinese Food Pagoda Score (CFPS) to measure the dietary quality of Chinese rural residents following recent research [17]. We found that Internet use significantly contributed to the dietary quality of rural adults, whose CFPS significantly improved by about 10.4% compared to their mean value. After resolving the potential endogeneity, our results remain robust. The effects of Internet use on the diet quality score for each food group as well as their actual intake amount were also investigated. For the former, the Internet use significantly increased the dietary quality score for five food groups: fruits, nuts, aquatic products, eggs, and salt. For the latter, Internet use significantly increased the consumption amounts of milk and its products (4 g), fruits (31.4 g), eggs (7.7 g), and vegetables (33.7 g), while the intake of oil (6 g) and salt (1.9 g) decreased.

The possible impact mechanism is that Internet use enriches rural residents’ information channels and promotes their dietary quality, which have been proved to have a positive effect on diet quality [45]. We found that the use of the Internet could significantly improve the dietary knowledge score of rural residents by 0.50, and the probability of knowing about the Chinese dietary pagoda was increased by 40%, which helps rural residents make better decisions in food consumption and promote dietary quality.

Furthermore, this paper demonstrates that there is heterogeneity in the effects of the Internet use on the dietary quality. The effect of Internet use was greater among females. This finding is consistent with the fact that women are more likely to seek online health information than men [37]. Rural residents who prepare food for the family as well as rural residents without college degrees benefited more from the use of the Internet.

Some interesting policy implications can be proposed according to the above findings. First, given that Internet use can effectively improve the dietary quality of rural residents, the Chinese government should continuously invest the Internet infrastructure to provide better Internet accessibility and information services, especially in the rural area. Considering that the China’s GDP and Internet development in 2011 is close to the current level of most developing countries (Appendix A Figure A1), this implication also has important reference value for many developing countries. Second, it is likely that giving information treatment to female and the person who prepare food for their family through Internet is an effective way to improve the dietary quality and national health, due to the heterogeneity in the impact of Internet use on dietary knowledge. Finally, although the Internet use has a positive effect on rural residents’ dietary quality, this also would increase their access to unhealthy food such as sugared drinks. Therefore, policymakers should pay attention to the negative externality of the Internet use and formulate effective strategies to guide residents to eat healthy.

There are some limitations to our study and we propose several implications for the further study. First, due to data limitation, we can only evaluate the short-term impact of Internet use on rural residents’ dietary quality, and the interpretation of our results should be based on the period from 2006 to 2011. Therefore, whether there are long-term effects of Internet use on dietary quality should be further examined, especially in the context of the rapid development of Information and Communication Technology. Second, the Internet may also affect people’s dietary quality through some other mechanisms, such as enhancing food accessibility [46] or increasing farmers’ production diversity [47], but we did not validate these mechanisms due to data limitations. The Internet use mainly through which mechanism to affect rural residents’ dietary quality need to be further discussed. In addition, since the Internet plays an important role in information transmission, it is worth further study on how policymakers should design effective information interventions with Internet against the sharp rise of rural obesity rates. Third, this study mainly focused on the effect of the Internet use on macro food intake, however, micronutrients (e.g., vitamins and minerals) also play an important role in human health [7,48]. In follow-up studies, the intake of micronutrients should also be considered in the evaluation system of diet quality, so as to more comprehensively assess the relationship between Internet use and diet quality. Finally, the Internet use effect on children and elderly’s dietary quality as well as other health behaviors is worthy of further investigation, since both the children and elderly are regarded as the group with a high risk of unbalanced nutrition intake [49,50].

## 5. Conclusions

In conclusion, based on three waves of CHNS panel data from 2006 to 2011, we find that the Internet use plays an important role in improving the dietary quality of China’s rural residents through increasing their knowledge about healthy diets. Furthermore, our study reveals that the positive effect of Internet use is greater among females, persons who cook for their families, and residents without a college degree. Therefore, our study confirms that Internet use plays an important role in improving the dietary quality of rural adults, and the government should pay more attention to constructing Internet infrastructure and promoting the popularization of the Internet.

## Figures and Tables

**Table 1:** Descriptive statistics of the variables.

Variable	Definition	Mean	Std. Dev.
CFPS	Chinese Food Pagoda Score	3.19	1.18
Internet	Whether use the Internet? Yes = 1, No = 0	0.07	0.25
Age	Responder’s age	45.34	8.91
Edu.	Education level, 0 = Illiterate; 1 = Primary school; 2 = Junior high school; 3 = High school; 4 = Secondary specialized school; 5 = Junior college or university; 6 = Postgraduate or above	1.74	1.20
log(hhincome)	Annual household income (log-form)	8.92	0.95
Household size	Total household number	4.01	1.54
Activity intensity	1 = very light; 2 = light; 3 = moderate; 4 = heavy; 5 = very heavy	3.14	1.08
Refrigerator	Has a refrigerator? Yes = 1, No = 0	0.60	0.49
Pressure Cooker	Has a pressure cooker? Yes = 1, No = 0	0.51	0.50
Car	Has a pressure car? Yes = 1, No = 0	0.08	0.28
Moto	Has a pressure moto? Yes = 1, No = 0	0.48	0.50
Agri-work	Percentage of household members participating in agricultural production (%)	43.15	28.36
Workout	Percentage of household members who leave the home for work (%)	28.47	20.42
Pave road	Connected to paved roads? Yes = 1, No = 0	0.72	0.45
Num. free markets	Number of free markets in the village	2.88	5.65

**Table 2:** Impact of Internet use on dietary quality of rural Chinese residents.

Dependent var.	CFPS		
[Mean]	[3.19]		
	(1)	(2)	(3)
Internet	0.3677 ***	0.3400 ***	0.3326 ***
	(0.1170)	(0.1128)	(0.1126)
Age		−0.0746	−0.0758
		(0.0983)	(0.0987)
Age2		0.0010 *	0.0010 **
		(0.0005)	(0.0005)
Edu.		0.0387	0.0375
		(0.0359)	(0.0362)
log(hhincome)		0.0289	0.0279
		(0.0303)	(0.0300)
Household size		−0.1017 ***	−0.1015 ***
		(0.0210)	(0.0211)
Activity intensity		−0.0052	−0.0072
		(0.0179)	(0.0182)
Refrigerator		0.1081 *	0.1126 *
		(0.0612)	(0.0614)
Pressure Cooker		−0.0239	−0.0247
		(0.0810)	(0.0809)
Car		−0.1213	−0.1210
		(0.0863)	(0.0868)
Moto		0.0066	0.0052
		(0.0423)	(0.0424)
Agri-work		0.0048 ***	0.0051 ***
		(0.0015)	(0.0015)
Workout		−0.0004	−0.0006
		(0.0015)	(0.0016)
Pave road			0.0027
			(0.0383)
Num. free markets			0.0064 **
			(0.0031)
Individual FE	Yes	Yes	Yes
Year FE	Yes	Yes	Yes
Observations	3607	3607	3607

Notes: ***, **, and * indicate significance at the 1%, 5%, and 10% levels, respectively. Robust standard errors are in parentheses and clustering is at the household ID.

**Table 3:** Internet use effects on dietary quality scores of rural adults for various food groups.

Dependent Var.	Milk and Its Products_ Score	Fruits_ Score	Legumes and Nuts_ Score	Meat and Poultry_ Score	Aquatic Products_ Score
[Mean]	[0.003]	[0.08]	[0.34]	[0.43]	[0.18]
	(1)	(2)	(3)	(4)	(5)
Internet	0.0003	0.1006 **	0.0246	0.0805 **	−0.0502
	(0.0055)	(0.0397)	(0.0506)	(0.0300)	(0.0439)
Control Variables	Yes	Yes	Yes	Yes	Yes
Individual FE	Yes	Yes	Yes	Yes	Yes
Year FE	Yes	Yes	Yes	Yes	Yes
Observations	3607	3607	3607	3607	3607
**Dependent Var.**	**Eggs_ Score**	**Cereals, Potatoes, and Beans_ Score**	**Vegetables_ Score**	**Oil_ Score**	**Salts_ Score**
**[Mean]**	**[0.24]**	**[0.37]**	**[0.42]**	**[0.62]**	**[0.54]**
	**(6)**	**(7)**	**(8)**	**(9)**	**(10)**
Internet	0.0791 **	0.0256	0.0514	0.1518 ***	0.1045 **
	(0.0328)	(0.0264)	(0.0461)	(0.0200)	(0.0416)
Control Variables	Yes	Yes	Yes	Yes	Yes
Individual FE	Yes	Yes	Yes	Yes	Yes
Year FE	Yes	Yes	Yes	Yes	Yes
Observations	3607	3607	3607	3607	3607

Notes: *** and ** indicate significance at the 1% and 5%, respectively. Robust standard errors are in parentheses and clustering is at the household ID.

**Table 4:** Internet use effects on food consumption amounts of rural adults for various food groups.

Dependent Var.	Milk and Its Products	Fruits	Legumes and Nuts	Meat and Poultry	Aquatic Products
[Mean]	[1.93]	[43.25]	[46.84]	[71.80]	[19.03]
	(1)	(2)	(3)	(4)	(5)
Internet	3.9745 *	31.3773 *	−11.3289	−9.7582	−5.3423
	(2.1201)	(16.4657)	(7.2369)	(8.1868)	(4.0659)
Control Variables	Yes	Yes	Yes	Yes	Yes
Individual FE	Yes	Yes	Yes	Yes	Yes
Year FE	Yes	Yes	Yes	Yes	Yes
Observations	3607	3607	3607	3607	3607
**Dependent Var.**	**Eggs**	**Cereals, Potatoes, and Beans**	**Vegetables**	**Oil**	**Salts**
**[Mean]**	**[25.27]**	**[414.75]**	**[281.04]**	**[33.86]**	**[8.69]**
	**(6)**	**(7)**	**(8)**	**(9)**	**(10)**
Internet	7.6571 **	−5.0877	33.6832 ***	−6.1190 **	−1.8749 **
	(3.1644)	(17.9368)	(8.3880)	(2.8298)	(0.8694)
Control Variables	Yes	Yes	Yes	Yes	Yes
Individual FE	Yes	Yes	Yes	Yes	Yes
Year FE	Yes	Yes	Yes	Yes	Yes
Observations	3607	3607	3607	3607	3607

Notes: ***, **, and * indicate significance at the 1%, 5%, and 10% levels, respectively. Robust standard errors are in parentheses and clustering is at the household ID.

**Table 5:** Mechanism test: Internet use, diet knowledge, and diet quality.

Dependent Var.	DKS	Knew about Chinese Dietary Pagoda (Yes = 1, No = 0)	CFPS	CFPS
[Mean]	[4.29]	[0.11]	[3.09]	[3.09]
	(1)	(2)	(3)	(4)
Internet	0.4980 *	0.4020 ***		
	(0.2613)	(0.0941)		
DKS			0.0127 ***	
			(0.0043)	
Knew about Chinese dietary pagoda				0.1877 **
				(0.0648)
Control Variables	Yes	Yes	Yes	Yes
Individual FE	Yes	Yes	Yes	Yes
Year FE	Yes	Yes	Yes	Yes
Observations	3607	3398	3607	3398

Notes: ***, **, and * indicate significance at the 1%, 5%, and 10% levels, respectively. Robust standard errors are in parentheses and clustering is at the household ID.

**Table 6:** Heterogeneous effects of Internet use on diet quality.

Dependent Var.	CFPS			
[Mean]	[3.19]			
	(1)	(2)	(3)	(4)
Internet	0.4703 ***	0.7676 ***	0.5980 *	0.4881 ***
	(0.1536)	(0.2162)	(0.2846)	(0.1270)
Internet × Female	0.2734 *			
	(0.1523)			
Internet × Cook		0.5163 **		
		(0.2468)		
Internet × High edu			−0.3915 *	
			(0.1847)	
Internet × High income				−0.3059
				(0.2606)
Control Variables	Yes	Yes	Yes	Yes
Individual FE	Yes	Yes	Yes	Yes
Year FE	Yes	Yes	Yes	Yes
Observations	3607	3607	3607	3607

Notes: ***, **, and * indicate significance at the 1%, 5%, and 10% levels, respectively. Robust standard errors are in parentheses and clustering is at the household ID.

**Table 7:** Impact of Internet use on dietary quality of rural Chinese residents: results of 2SLS method.

2nd Stage Regression	Dependent Var.: CFPS	
[Mean]	[3.19]	
	(1)	(2)
Internet	0.8994 *	0. 7861 ***
	(0.5227)	(0.1751)
Control variables	No	Yes
Individual FE	Yes	Yes
Year FE	Yes	Yes
**1st Stage Regression**	**Dependent Var.: Internet**	
Computer (Yes = 1, No = 0)	0.1098 ***	0.1101 ***
	(0.0132)	(0.0134)
Control variables	No	Yes
Individual FE	Yes	Yes
Year FE	Yes	Yes
F-value	17.72	13.52
Observations	3607	3607

Notes: *** and * indicate significance at the 1% and 10% levels, respectively. Robust standard errors are in parentheses and clustering is at the household ID.

**Table 8:** Impact of Internet use on other health-related food intake: sugared drinks and alcohol.

Dependent Var.	Sugared Drinks Consumption Frequency	Weekly Beer Consumption	Weekly Liquor Consumption
[Mean]	[0.71]	[0.55]	[2.36]
	(1)	(2)	(3)
Internet	0.3359 *	−0.1666	−0.1400
	(0.1983)	(0.2383)	(0.7870)
Control Variables	Yes	Yes	Yes
Individual FE	Yes	Yes	Yes
Year FE	Yes	Yes	Yes
Observations	3597	3600	3599

Notes: * indicates significance at 10% levels. Robust standard errors are in parentheses and clustering is at the household ID. “Sugared drinks consumption frequency” is expressed by numbers 0–5, with “0” as “never drink”, “1” as “drink less than once monthly”, “2” as “1–3 times monthly”, “3” as “once or twice weekly”, “4” as “3–4 times weekly”, and “5” as “everyday”. Unit of weekly beer consumption is “bottle (500 g)”. Unit of weekly liquor consumption is “liang (50 g)”.

## Data Availability

The raw data used in this research can be downloaded directly from the China Health and Nutrition Survey (https://www.cpc.unc.edu/projects/china/data/datasets, access on 1 March 2022).

## Appendix A

**Table A1:** Chinese Food Pagoda Score (CFPS) for various calorie intakes.

Food Group	Guideline	1600 kcal	1800 kcal	2000 kcal	2200 kcal	2400 kcal	2600 kcal	2800 kcal
	(1)	(2)	(3)	(4)	(5)	(6)	(7)	(8)
*Cereals*, *Potatoes*, and *Beans*								
Score “1”	250–400	175–225	200–250	225–275	250–300	275–325	325–375	350–400
Score “0.5”		88–175	100–200	113–225	125–250	138–275	163–325	175–350
Score “0.5”		225–338	250–375	275–413	300–450	325–488	375–563	400–600
*Aquatic Products*								
Score “1”	40–75	≥40	≥50	≥50	≥75	≥75	≥75	≥100
Score “0.5”		20–40	25–50	25–50	38–75	38–75	38–75	50–100
*Eggs*								
Score “1”	40–75	40–75	40–75	40–75	40–75	40–75	40–75	40–75
Score “0.5”		20–40	20–40	20–40	20–40	20–40	20–40	20–40
Score “0.5”		50–75	50–75	50–75	50–75	50–75	50–75	50–75
*Milk* and *Its Products*								
Score “1”	300	≥300	≥300	≥300	≥300	≥300	≥300	≥300
Score “0.5”		150–300	150–300	150–300	150–300	150–300	150–300	150–300
*Fruits*								
Score “1”	200–350	≥200	≥200	≥300	≥300	≥350	≥350	≥400
Score “0.5”		100–200	100–200	150–300	150–300	175–350	175–350	200–400
*Meat* and *Poultry*								
Score “1”	40–50	15–65	25–75	25–75	50–100	50–100	50–100	75–125
Score “0.5”		8–15	13–25	13–25	25–50	25–50	25–50	38–75
Score “0.5”		65–98	75–113	75–113	100–150	100–150	100–150	125–188
*Vegetables*								
Score “1”	300–500	≥300	≥400	≥450	≥450	≥500	≥500	≥500
Score “0.5”		150–300	200–400	225–450	225–450	250–500	250–500	250–500
*Legumes* and *Nuts*								
Score “1”	25	15–25	15–25	15–25	25–35	25–35	25–35	25–35
Score “0.5”		8–15	8–15	8–15	13–25	13–25	13–25	13–25
Score “0.5”		25–38	25–38	25–38	35–53	35–53	35–53	35–53
*Oil*								
Score “1”	25–30	≤25	≤30	≤30	≤30	≤30	≤30	≤30
Score “0.5”		25–38	30–45	30–45	30–45	30–45	30–45	30–45
*Salts*								
Score “1”	6	≤6	≤6	≤6	≤6	≤6	≤6	≤6
Score “0.5”		6–9	6–9	6–9	6–9	6–9	6–9	6–9

Notes: The calorie intake level is the upper bound for each interval. For example, individuals with a calorie intake lower than or equal to 1600 kcal are regarded as falling within the 1600 kcal group.

**Table A2:** Diet knowledge.

Do You Strongly Agree, Agree, Are Neutral About, Disagree, or Strongly Disagree with this Statement?	True or False
Choosing to eat more fresh fruits and vegetables is good for your health	TRUE
Eating more sugar is good for your health	FALSE
Eating different kinds of food is good for your health	TRUE
A high-fat diet is good for your health	FALSE
Choosing a diet with more staple foods (rice products, wheat products) is not good for your health	TRUE
Eating many animal products (fish, poultry, eggs, lean meat) every day is good for your health	FALSE
Reducing fat and animal fat in the diet is good for your health	TRUE
Drinking milk and its products is good for your health	TRUE
Eating beans and its products is good for your health	TRUE
